# Changes in the net primary production of ecosystems across Western Europe from 2015 to 2022 in response to historic drought events

**DOI:** 10.1186/s13021-024-00279-9

**Published:** 2024-09-18

**Authors:** Christopher Potter, Stephanie Pass

**Affiliations:** 1 NASA Ames Research Center, Moffett Field, CA USA; 2 University of California, Berkeley, CA USA

**Keywords:** Carbon, Ecosystems, Net primary production, Drought, MODIS, Landsat

## Abstract

**Background:**

Ecosystem models are valuable tools to make climate-related assessments of change when ground-based measurements of water and carbon fluxes are not adequately detailed to realistically capture geographic variability. The Carnegie-Ames-Stanford Approach (CASA) is one such model based on satellite observations of monthly vegetation cover to estimate net primary production (NPP) of terrestrial ecosystems.

**Results:**

CASA model predictions from 2015 to 2022 for Western Europe revealed several notable high and low periods in growing season NPP totals in most countries of the region. For the total land coverage of France, Greece, Italy, Portugal, and Spain, 2018 was the year with the highest terrestrial plant growth, whereas 2017 and 2019 were the years with the highest summed NPP across the UK, Germany, and Croatia. For most of Western Europe, 2022 was the year predicted with the lowest summed plant growth. Annual precipitation in most countries of Western Europe gradually declined from a high average rate in 2018 to a low average precipitation level in 2022.

**Conclusions:**

The CASA model predicted decreased growing season NPP of between − 25 and − 60% across all of Spain, southern France, and northern Italy from 2021 to 2022, and much of that plant production loss was detected in the important cropland regions of these nations.

## Plain language summary

Annual growth of terrestrial plant cover, also known as net primary production (NPP), must be maintained and managed by societies worldwide to meet their essential needs for food and fiber. A model of global NPP that uses monthly satellite images as inputs predicted that extreme drought in the years 2020 and 2021 across Western Europe, caused by greatly diminished precipitation totals and elevated temperatures, depressed plant production by between − 25 and − 60% compared to the previous five years in France, Italy, and Spain.

## Background

Net photosynthetic accumulation of carbon by plants, also known as net primary production (NPP), supports most biotic processes on Earth [1, 2]. NPP is cultivated in agricultural systems to provide the food and fiber required by human populations [3]. Nonetheless, drought and extreme heat, together or separately, can have disruptive effects on the carbon cycles of terrestrial ecosystems, and particularly in summer-dry forests and savannas [4].

In Western Europe, numerous extreme weather events and wildfires over the past decade have caused major disturbance incidents and billions of dollars in damage to agricultural yields and to water and energy supplies [5]. The annual cost of drought for the European Union and the United Kingdom (EU + UK) has been estimated at between 9 and 20 billion EUR [6]. Weather-related hardships over the past several years that stand-out include: the River Rhine’s water levels through Germany have fallen to levels that shipping was disrupted; more than 100 cities in France have run out of drinking water; major wildfires have burned in Italy, France, and Greece in 2021. In years of abundant precipitation, there is frequently major flooding along the Ebro River in northern Spain, causing widespread damage to agriculture and livestock. For instance, in December 2021, inundation in the river’s floodplain affected 50,000 hectares, including 14,000 ha of cropland [7].

As characterized by Tripathy and Mishra [5], the Compound Drought and Heatwave (CDHW) of 2022 in Europe resulted in widespread crop failures, water deficits, river drying, and wildfires, with the greatest disruption to the environment on the Iberian Peninsula, southern France, and northern Italy. In these countries, air surface temperatures were 2.5 °C above normal, and extreme drought conditions lasted from May to August of 2022. Around 55% of the European land mass experienced severe drought during 2022 and as much as to 70% suffered through high-temperature anomalies (> 1 °C), with up to 50% and 20% of the area experiencing anomalies > 2° and 3 °C, respectively. This study further estimated that the historical return period for another 2022 CDHW event ranged between 280 and 420 years, making this event unprecedented in the modern European era.

Northern Italy experienced severe water shortages and decreased hydropower energy production during summer of 2022, as confirmed by the Po River Basin Authority [8]. Drainages in the Po River basin most strongly impacted were reported around Piacenza, distinguished by a 66% deficit in river discharge rates and sea water intrusion into the Po River Delta, comparable to the most critical previous levels experienced in the summer of 2003 [9]. These authors also reported that low snow accumulation (of only around 40% of the 2009–2021 median levels) in the southern Alps was recorded during the winter of 2022.

Based on eddy-covariance and meteorological data, along with ecosystem carbon modeling at 14 forest sites in Europe, van der Woude et al. [10] reported reduced forest carbon uptake during the 2022 drought and heat waves. Sites in southern France showed widespread summertime carbon release by forests, in addition to large carbon dioxide (CO_2_) emissions from wildfires. Severe droughts in 2018 and 2022, with record moisture deficits in the summer months, have raised the level of uncertainty for the future of a European forest sink for atmospheric CO_2_.

To help resolve these types of uncertainties, the CASA (Carnegie-Ames-Stanford Approach) carbon cycle model [1, 11] can estimate the monthly NPP flux of atmospheric CO_2_ between plants and soils on a global scale using satellite image inputs from the NASA Moderate Resolution Imaging Spectroradiometer (MODIS). CASA is the only global carbon model that has consistently used MODIS and Landsat products for land cover classes and green vegetation indices as monthly inputs to drive the prediction of NPP and soil CO_2_ emissions in the terrestrial biosphere. It is the most well-integrated model of the global carbon and water cycles with high-level products from NASA satellite remote sensing missions. Moreover, the nominal 8-km grid cell resolution of the CASA model enables localized studies of ecosystem carbon and water fluxes of interest to public sector stakeholders working at nearly every organizational level from local to regional.

Recently, the CASA model has been a cornerstone of science investigations to evaluate results of CO_2_ fluxes from NASA’s Orbiting Carbon Observatory (OCO-2 and OCO-3) mission, as illustrated in the publication by Philip et al. [12]. These OCO-2 inverse model results for validating observed CO_2_ patterns in the atmosphere used CASA outputs as prior conditions for the land surface CO_2_ flux contribution. The CASA model is also the foundation for the CASA-GFED (Global Fire Emissions Database) model, which estimates monthly NPP and soil heterotrophic respiration globally, along with biomass burning emissions of CO_2_ each year [13].

CASA NPP model calibration has been validated repeatedly, first globally by comparing predicted annual NPP to more than 1900 field measurements of NPP [1, 11]. Interannual NPP fluxes from the CASA model have been reported [14] and validated against multiyear estimates of NPP from field stations and tree rings [2]. The CASA model has been validated against field-based measurements of ecosystem CO_2_ fluxes and carbon pool sizes at multiple boreal forest sites in Western Europe [15–17] and against atmospheric inverse model estimates at the global scale [18]. More recently, Jay et al. [3] validated CASA NPP estimates using 17 Ameriflux tower flux sites located across North America.

In the present study, the CASA model has been applied to the Western European region over the period of 2015 to 2022. The primary research questions posed in this study were:

- How have variations in precipitation and air temperature impacted NPP in all ecosystems of Western Europe over the study period?
- How have recent changes in NPP been distributed across the major river basins and cropping systems of southern Europe?

We focused on the main growing season months of May to August to more precisely isolate and evaluate the impacts of variable precipitation totals and extreme heat events on plant production. CASA model outputs were summed and averaged for growing season NPP (g C m^− 2^) for all countries of Western Europe from 2015 to 2022. The results presented begin at the large scale of the continent and zoom down to individual crop fields in southern Europe that were heavily impacted by recent droughts.

## Methods

### CASA NPP algorithms

Monthly net vegetation carbon fixation patterns, or NPP, were computed from the CASA model by using the concept of light-use efficiency [19]. NPP is calculated as a product of time-varying solar radiance (S_r_), precipitation, air temperature, the Enhanced Vegetation Index (EVI) from MODIS satellite imagery, and a constant light utilization efficiency term (*e*_*max*_) that is modified by time-varying stress terms for temperature (T) and moisture (W) [20].

$$\:NPP\:=\:{S}_{r}\:EVI{\:e}_{max}\:T\:W$$

Based on calibration using field estimates of NPP from across the globe, the constant *e*_*max*_ term was set at 0.55 C MJ^− 1^ S_r_ [1, 11].

The air temperature (T) stress scalar for CASA NPP computation is computed with reference to derivation of optimal temperatures (Topt) for plant production. The Topt setting will vary by latitude and longitude, ranging from near 0 °C in the Arctic to the middle thirties in low-latitude deserts. For this study, Topt has been updated using air temperatures for the past several years (2015–2022), to best reflect the recent trends in climate warming. The W stress scalar is estimated from monthly water deficits, based on a comparison of moisture supply (precipitation and stored soil water) to potential evapotranspiration (PET) demand using the method of Priestly and Taylor [21].

Evapotranspiration is connected to water content in the CASA model soil profile layers, as estimated using the algorithms described by Potter [20]. The soil model design includes three-layer (M1–M3) heat and moisture content computations: surface organic matter (SOM), topsoil (0.3 m), and subsoil to rooting depth (1–2 m). These layers can differ in soil texture, moisture holding capacity, and carbon–nitrogen dynamics. Water balance in the soil is modeled as the difference between precipitation or volumetric percolation inputs, monthly estimates of PET, and the drainage output for each layer. Inputs from rainfall can recharge the soil layers to field capacity. Excess water percolates through to lower layers and may eventually leave the system as seepage and runoff. Freeze–thaw dynamics with soil depth operate according to the empirical degree-day accumulation method [22], as described by [23].

### Global input datasets

#### NCEP

Global monthly data from the NCEP-DOE Reanalysis 2 dataset was acquired for the years 2015 to 2022 via the National Oceanic and Atmospheric Administration (NOAA) data portal [24]. Monthly mean air temperatures, air maximum temperatures, air minimum temperatures, mean solar radiation flux, and mean precipitation rate files were acquired for CASA model inputs.

In order to prepare the NCEP data for the CASA model, unit conversions were necessitated. First, all of the NCEP files were reprojected into the Mollweide (ESRI:54009) spatial reference system with 8-km size cells and passed through a 20 × 20 focal smoothing filter. NCEP temperature values were converted from degrees kelvin to Celsius. Solar radiation flux was converted from watts per square meter (W m^2^) to Megajoules (MJ mo^− 1^), taking into account average daylight minutes per month for Western Europe. Precipitation values were converted from kilograms m^− 2^ to cm mo^− 1^.

#### MODIS EVI

Terra MODIS data sets for the years 2015 to 2022 were obtained from NASA’s Land Processes Distributed Active Archive Center site (LP-DACC) [25]. One 16-day Enhanced Vegetation Index (EVI) file was chosen for each month from the MOD13C1 Version 6 data repository to obtain CASA input data. The global composite (cloud-adjusted) MODIS imagery was converted to 8-km resolution and into a Mollweide spatial reference system.

#### MODIS land cover

The MODIS 1-km land cover map [26] was aggregated to 8-km pixel resolution and used to specify the predominant land cover class. These classes were used to assign the soil rooting depth settings in CASA as either forest, shrubland, or grassland [11].

### Statistical methods

Monthly NPP files for the years 2015 to 2022 were output from the CASA model. We selected the growing season (May through August) NPP files to further analyze with R [27]. The monthly NPP growing season values were summed into yearly values using the raster package in R [28]. Polygon shapefiles of county boundaries, major river basins, and land cover classes [29] were used to sub-divide the NPP gridded output files for further analysis. NPP growing season averages and sums were ascertained from the model output files using the raster package’s cellStats function [28]. The 8 × 8 km cell size was taken into account to obtain the total summed grams of carbon per country. A similar process was also carried out to obtain average yearly rainfall and yearly mean air temperature values from the monthly NCEP input files to the CASA model.

We used the quantile function from the stats package (version 4.2.2) in R [27] to calculate the quantiles around the mean for average growing season NPP, average yearly precipitation (cm y^− 1^), and average air temperature (^o^C) for selected countries in Western Europe. We used the 25th and 75th percentiles to identify the interquartile range (IQR) to plot and demonstrate the spread of the values for each country. These quantiles are represented as error bars for the plotted mean data for country-wide NPP, yearly mean precipitation, and yearly mean temperature. The R packages ggplot2 and ggmatplot [30] were used to visualize the data.

**Fig. 1:**
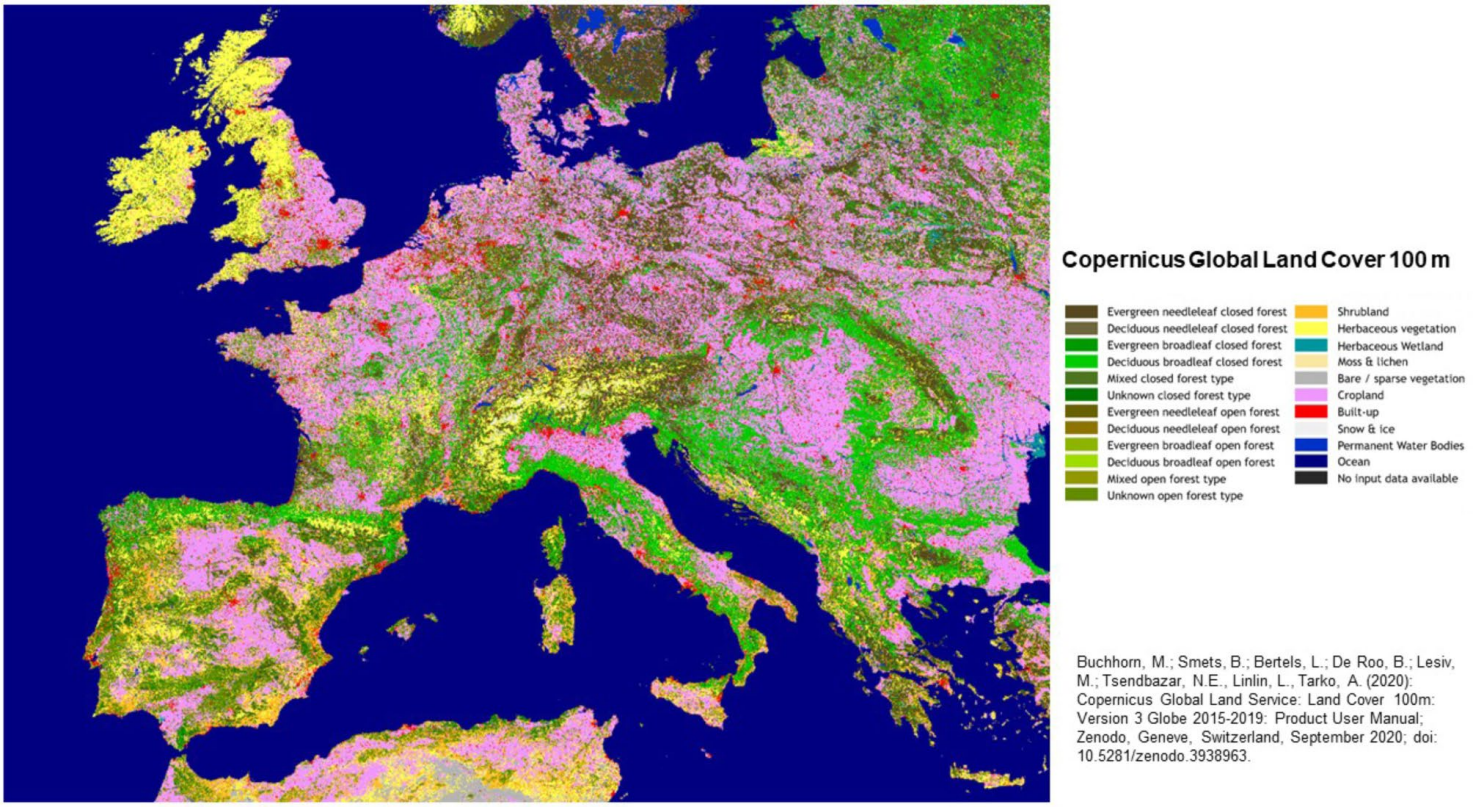
Land cover class map (100 m resolution) for Western Europe [29]

To visualize more fine-scale variations in predicted NPP within selected river basins, Landsat (30-m) EVI images for the growing season months of 2019 were used to downscale the 8 × 8-km gridded CASA model monthly outputs. The range of Landsat EVI (from 0 to 1.0) was rescaled within each 8 × 8 km grid cell area to generate a multiplier raster file with values ranging from around 0.1 to 1.5, with a value of 1.0 corresponding to the MODIS EVI value of each overlapping 8 × 8 km grid cell. This Landsat pixel resolution grid of values was multiplied by the CASA NPP growing season summed value for each year to downscale the NPP estimates to 30-m pixel sizes. Land cover classes mapped at 100-m resolution by the 2020 Copernicus Global Land Service [29] were used to compute statistics (mean, maximum, and variation) by major cover classes (Fig. 1).

## Results

### CASA NPP predictions 2015 to 2022

**Fig. 2:**
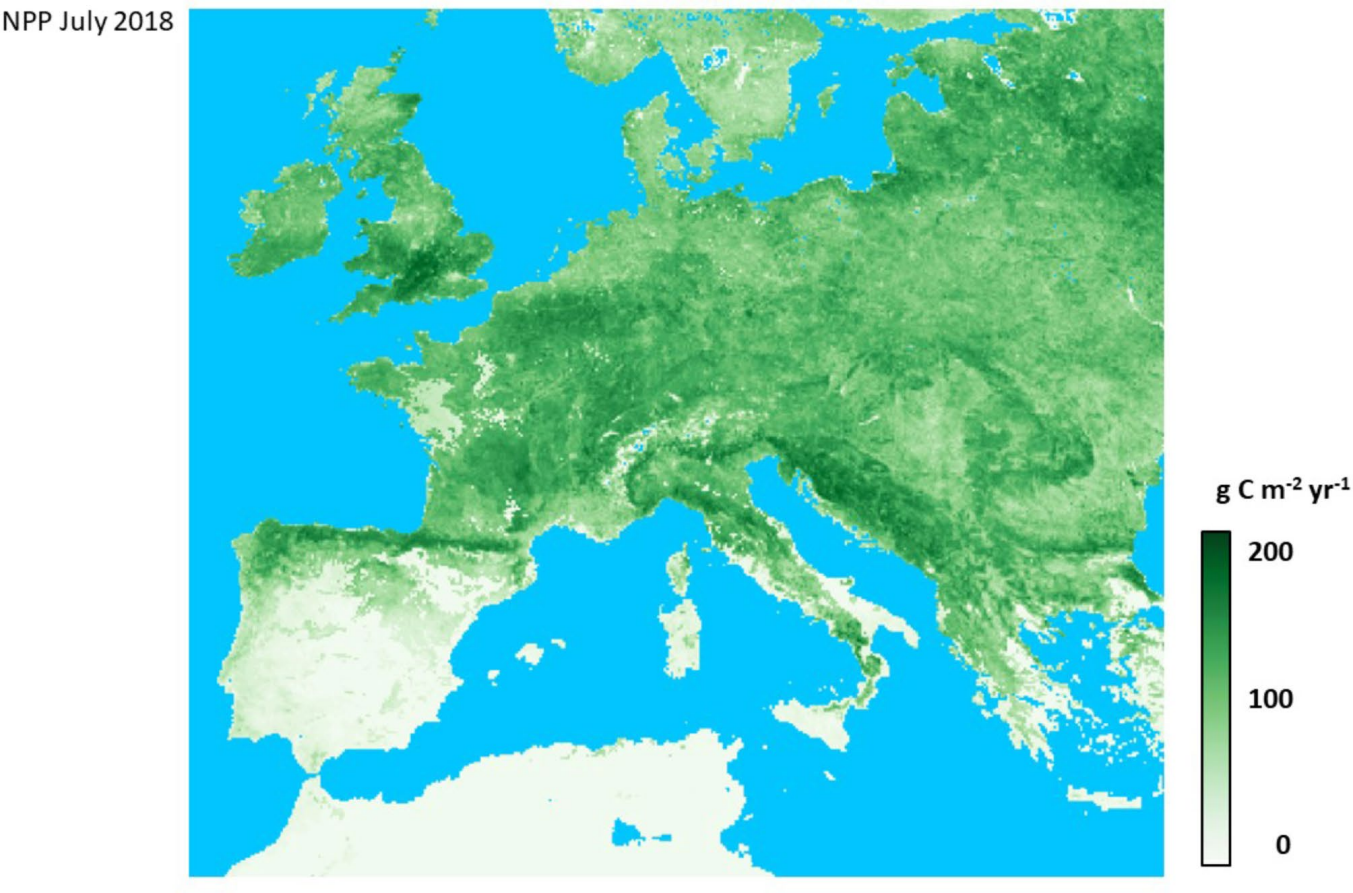
Map of CASA predicted NPP for July 2018 in Western Europe

The localized areas of Western Europe with the highest NPP levels predicted during the peak of summer growing seasons (Fig. 2) included: Cotswolds to North Wessex Downs in the UK; southeastern Belgium; woodlands north of Frankfurt, Germany; Mounts of Cantal in the Massif Central of France; Vosges and Jura ranges of eastern France; northern Pyrenees mountains; eastern Italian Alps around Lago di Garda; Apennines range of western Italy; Karlovac and Lika-Senj Counties in northern Croatia. The southern Mediterranean coastal areas and islands of Spain, Italy, and Greece were predicted with the lowest NPP levels during the peak of the summer growing season; these regions display peak plant production in the cooler spring months of the year instead of the hot summer months.

**Fig. 3:**
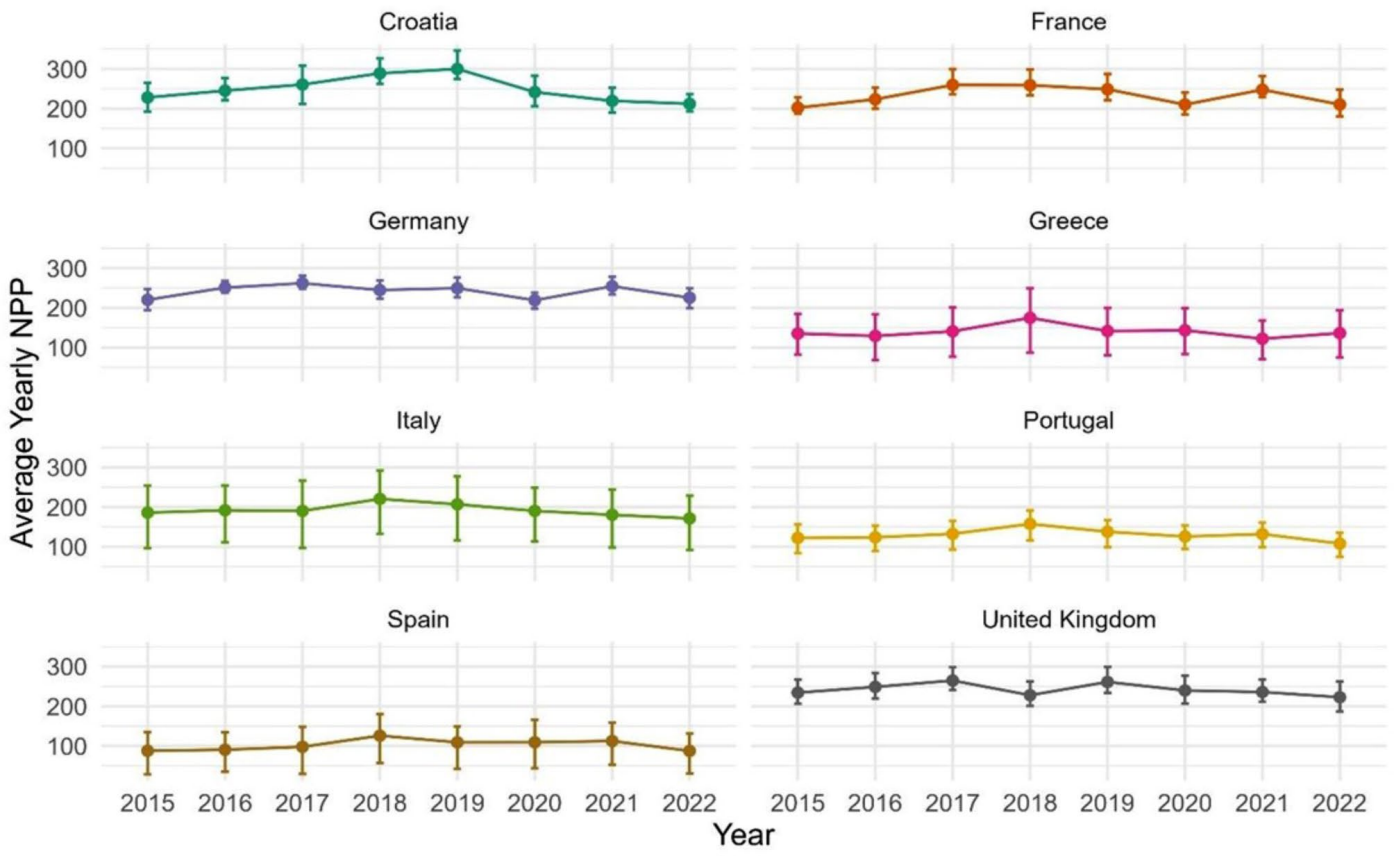
Average growing season NPP (g C m^− 2^) for selected Western European countries from 2015 to 2022 displayed with their interquartile range

Averaged on a country-wide basis, total growing season NPP (g C m^− 2^) from 2015 to 2022 was shown to be highest (in a range of 200–300 g C m^− 2^) for Germany, France, Croatia, and the United Kingdom (Fig. 3). These four countries also had the narrowest range (geographically) of growing season NPP values over their total land coverage within any given year. Greece, Italy, Portugal, and Spain typically each had predicted average growing season NPP from 2015 to 2022 in a lower range of 100–200 g C m^− 2^ and showed a relatively wider range of NPP values over their total land coverage within any given year.

Average growing season NPP declined markedly between 2021 and 2022 in Croatia, France, Germany, Italy, Portugal, and Spain (Fig. 3). Average plant production in Italy and Spain was predicted at relatively low levels during the growing seasons of 2016 and 2017 as well.

**Fig. 4:**
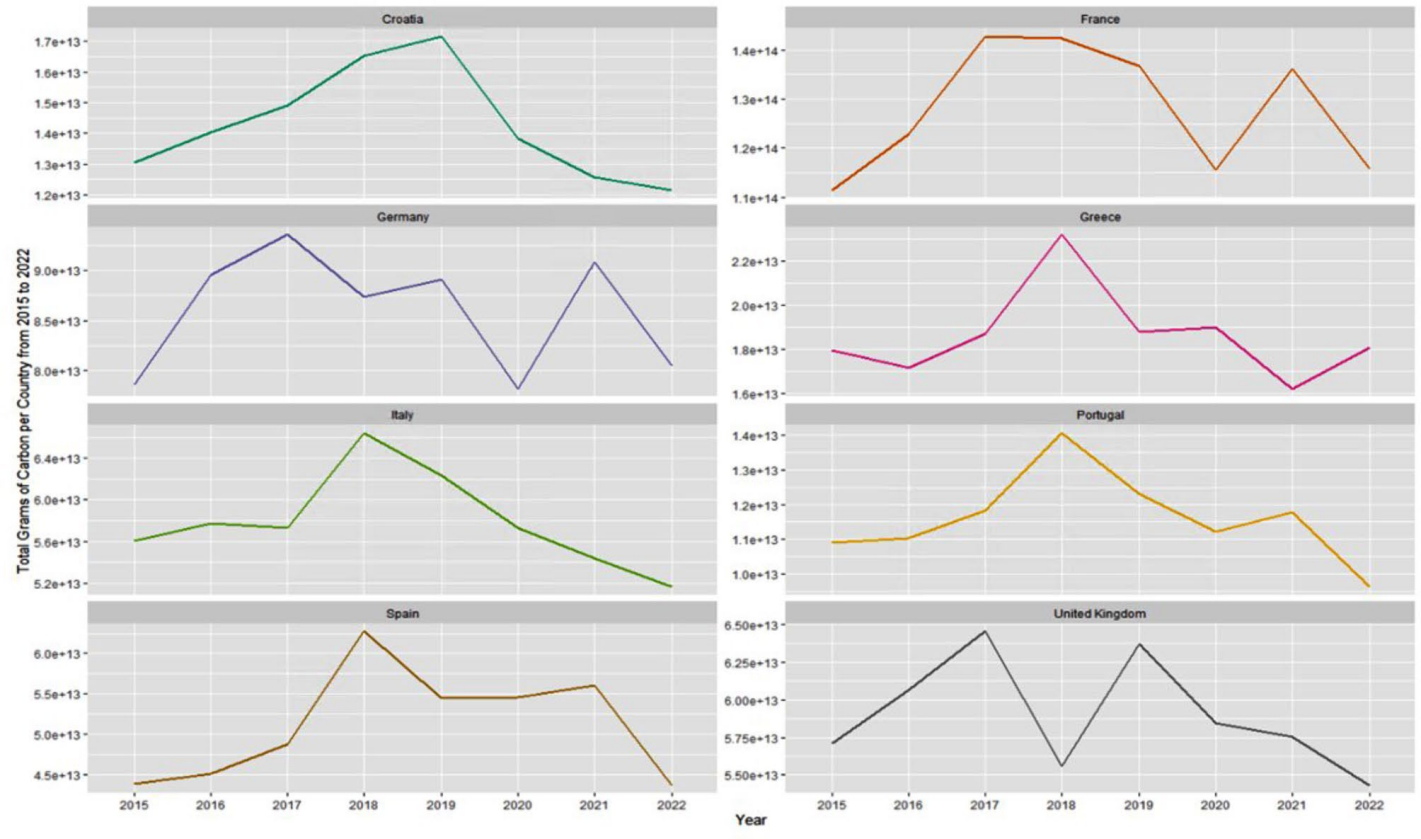
Comparison of summed growing season NPP (g C) in selected countries from 2015 to 2022

Over the study period of 2015 to 2022, several notable high and low periods were identified for growing season NPP sums (in total grams C) for most countries in western Europe (Fig. 4). Across the summed land coverage of France, Greece, Italy, Portugal, and Spain, 2018 was the year with the highest terrestrial plant growth over the study period. Across the UK, Germany, and Croatia, 2017 and 2019 were the years with the highest summed NPP during the study period. For most of these eight selected countries, 2022 was the year predicted with the lowest summed plant growth, the exceptions being Germany and Greece, for which 2022 was the second lowest year of plant growth in the time series.

### Precipitation and temperature variations 2015 to 2022

**Fig. 5:**
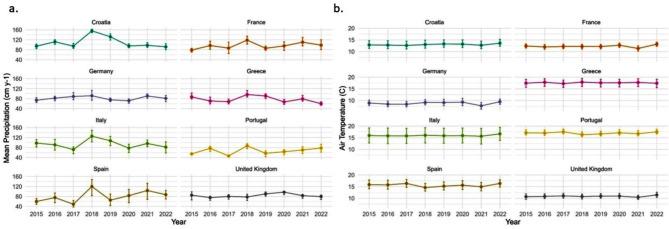
(**a**) Annual precipitation totals in cm y^− 1^, and (**b**) annual average air temperatures (degrees Celsius) for selected Western European countries from 2015 to 2022 displayed with their Interquartile Range

Annual precipitation in most countries of Western Europe progressively declined from a high average rate of greater than 90 cm y^− 1^ in 2018 to a low average precipitation rate of less than 80 cm y^− 1^ in 2022 (Fig. 5a). The exception was the UK, which received its highest precipitation rate in 2020 during the study period, instead of in 2018. Croatia, France, Italy, Portugal, and Spain all received relatively low precipitation amounts in 2017 as well. Croatia, Germany, and Greece were the countries with the consistently narrowest range of variation in precipitation rates across their respective land cover area. The countries with the consistently widest range of geographic variation in precipitation rates were Spain, France, and Italy, particularly in 2022.

Annual average air temperatures increased slightly over the study period of 2015 to 2022 in Western Europe, mainly owning to a continental-wide warming trend from 2021 to 2022 (Fig. 5b). The countries with the consistently widest range of geographic variation in surface temperatures across their land area coverage were Greece and Italy.

### CASA NPP response to extreme drought in 2022

**Fig. 6:**
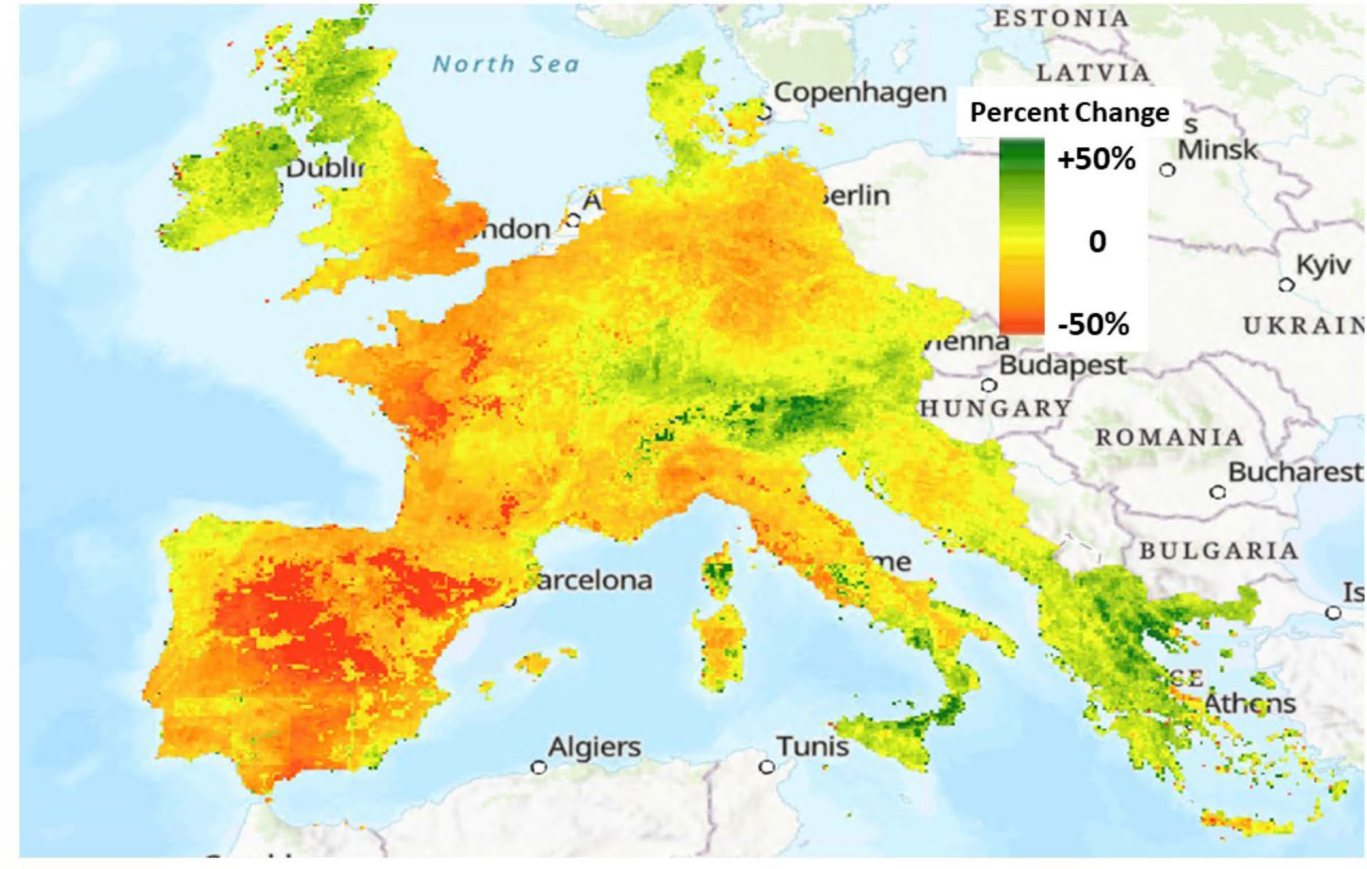
Change (in percent) in the summed growing season NPP from 2021 to 2022 across Western Europe

The relative change (in percent) of NPP from 2021 to 2022 (Fig. 6) across sub-regions of Western Europe was most notable across the following geographic coverages:

- A pattern of increased (+ 20%) to decreased (-35%) growing season NPP from Ireland across to eastern England from 2021 to 2022, respectively.
- A pattern of decreased (-40%) to increased (+ 10%) growing season NPP from the western Loire Valley of France across to the Vosges from 2021 to 2022, respectively.
- A pattern of decreased (between − 25 and − 40%) growing season NPP in the Elbe River basin of eastern Germany from 2021 to 2022,
- A pattern of decreased (between − 25 and − 60%) growing season NPP across all of Spain, southern France, and northern Italy and Croatia from 2021 to 2022,
- A pattern of increased (between + 30 and + 45%) growing season NPP across the eastern Alps and in North Macedonia and in eastern Greece from 2021 to 2022.

### CASA NPP at landsat resolution for major river basins

**Fig. 7:**
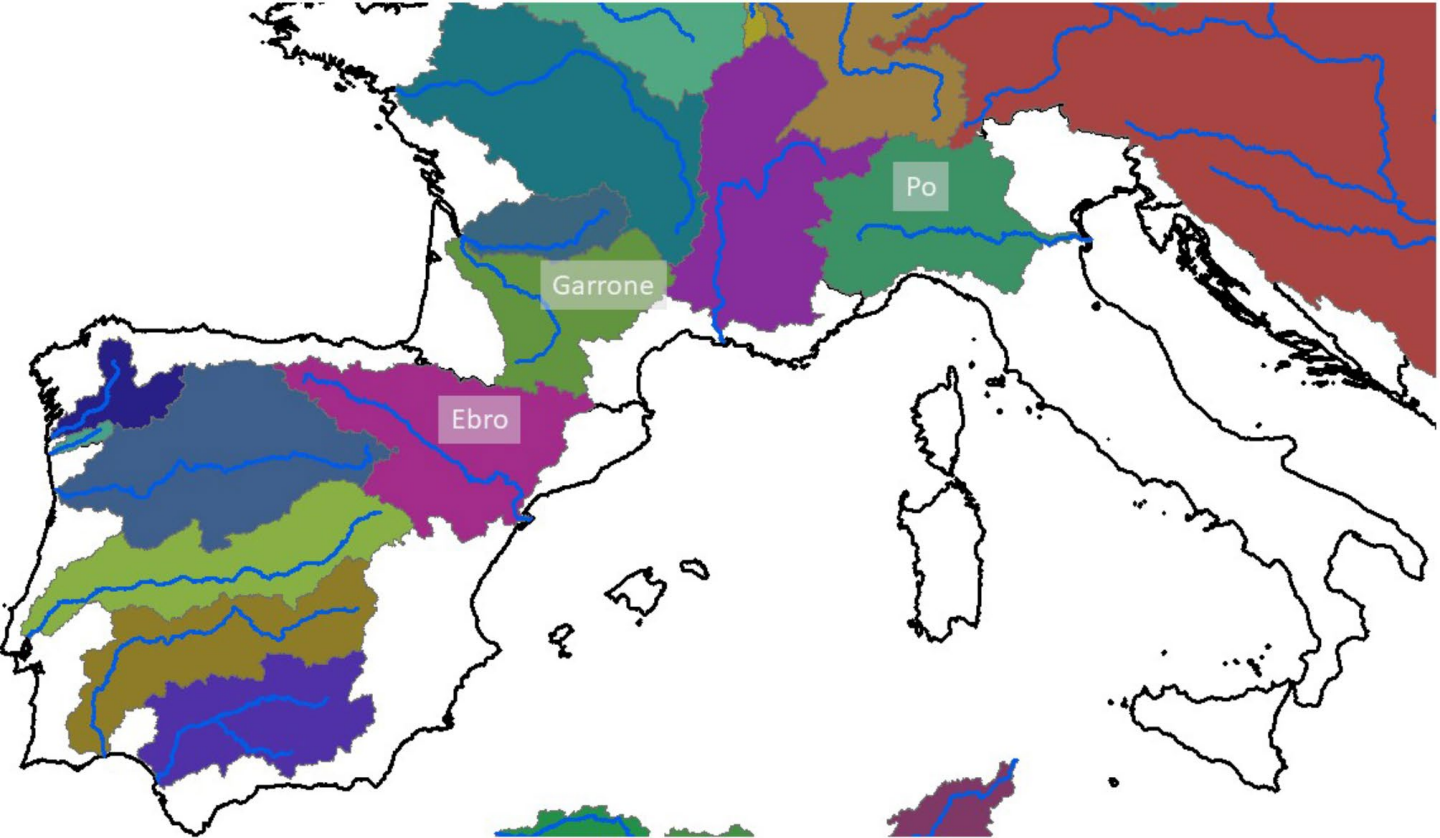
Location and extent of the Ebro, Garrone, and Po River basins in southern Europe

For a closer examination of drought impacts, three large river basins were selected that typified the pattern of decreased growing season NPP in excess of -50% from 2021 to 2022 in northern Spain, southern France, and northern Italy. These were the Ebro, Garrone, and Po River basins (Fig. 7). Land cover in these three river basins was predominantly croplands (33%) and open woodlands and shrublands (22%) [29].

For all three of these large river basins combined, the major land cover classes within which growing season NPP was most strongly impacted by drought conditions 2022 (compared to the 2020 and 2021 growing seasons) were shrubland at -27%, cropland at -25%, and open forest at -21% (Table 1). The maximum predicted growing season NPP in 2022 was reduced by 30% in these land cover classes. The variability of growing season NPP within the three river basins combined also contracted by 11–13% in 2022 compared to the two previous growing seasons.

**Table 1:** Change in growing season NPP from 2020/21 to 2022 predicted by the CASA model for land cover classes [29]. In the combined Ebro, Garrone, and Po River basins in Spain, France, and Italy, respectively

Land Cover Class			Area (km^2^)	% Change 2020/21 to 2022		
				Maximum	Mean	Standard Deviation
Shrubland			12,958	-30	-27	-13
Herbaceous vegetation			26,086	-30	-14	-12
Cropland			71,078	-30	-25	-12
Urban			8,982	-30	-21	-11
Permanent water bodies			1,463	-16	-13	-10
Herbaceous wetland			280	-34	-21	-14
Closed forest, evergreen needle leaf			11,719	-19	-13	-5
Closed forest, deciduous broad leaf			27,728	-19	-16	-15
Closed forest, mixed			4,326	-16	-9	9
Closed forest, unknown type			9,621	-23	-18	-7
Open forest, evergreen needle leaf			550	-9	-16	12
Open forest, deciduous broad leaf			2,407	-19	-18	-10
Open forest, mixed			1,490	0	-18	-7
Open forest, unknown type			34,627	-30	-21	-11

The Ebro River in the northern Iberian Peninsula flows 930 km into the Mediterranean Sea, forming a delta in the Province of Tarragona in southern Catalonia. The Ebro drainage area of 85,550 km^2^ is the largest in Spain. Nonetheless, its mean annual flow has decreased by approximately 29% during the 20th century due to construction of dams and the increasing demands for irrigation of croplands [31]. The main crops grown in the Ebro Valley include barley, wheat, fruit and olive orchards, vineyards, corn, and alfalfa [32].

**Fig. 8:**
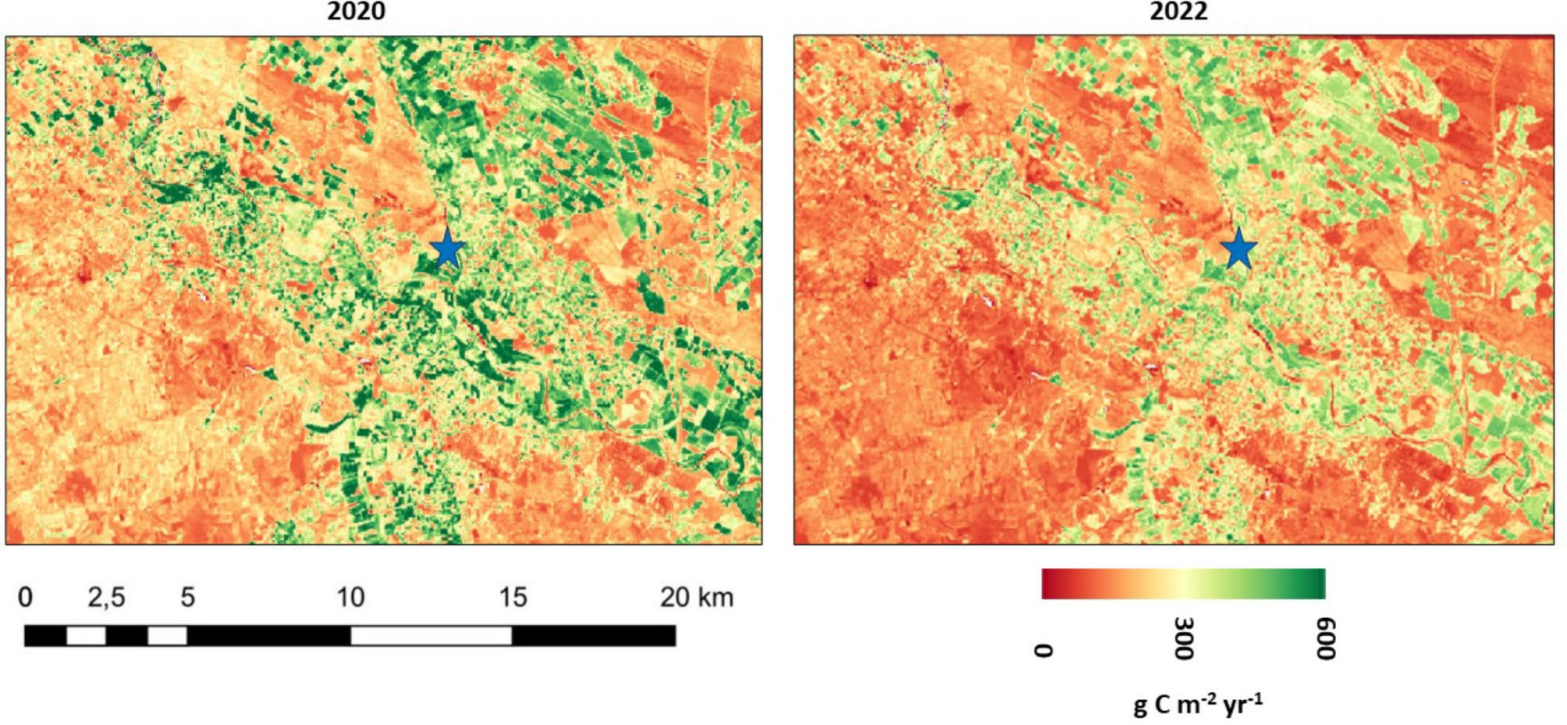
CASA model NPP at 30-m Landsat pixel resolution predicted for the growing seasons of 2020 (left) and 2022 (right) for the Ebro River basin drainages of the Aragon River to its north. Location of blue star symbol is 42.23^o^ N, 1.75^o^ W near the city of Milagro

Localities within the Ebro River basin (example in Fig. 8) where the 2020/2021 growing season NPP was reduced by 50% or more by drought conditions in 2022 included:

- Aragón-Arga River sub-basins, within which the main river course is dammed near Esteríbar to supply water to the Pamplona metropolitan area;
- Lower Gallego and Cinca sub-basins, flowing into the Ebro downstream of the city of Zaragoza where the historical discharge has been reduced to 10% of the natural rate upstream by diversions [33].
- Lower Martin River sub-basins, flowing from the Iberian Range and regulated by the Cueva Foradada dam near the localities of Alcaine and Oliete.

The Garonne River in southwest France and northern Spain flows 602 km from the central Pyrenees and the Massif Central to the Gironde estuary at the port of Bordeaux. Its watershed drains 55,000 km^2^ to the Atlantic Ocean. The forested slopes of the Pyrenees make up 35% of the watershed area. The alluvial aquifer of the Garonne is considered a large regional reservoir and an important irrigation source for the agricultural activities in the Garonne Valley [34]. In addition to supplying over two million people with drinking water, more than 60% of the Garonne Valley is used for growing crops and livestock, with around 15% of this farmland in irrigated fields devoted mainly to corn, sunflower, and wheat [35].

**Fig. 9:**
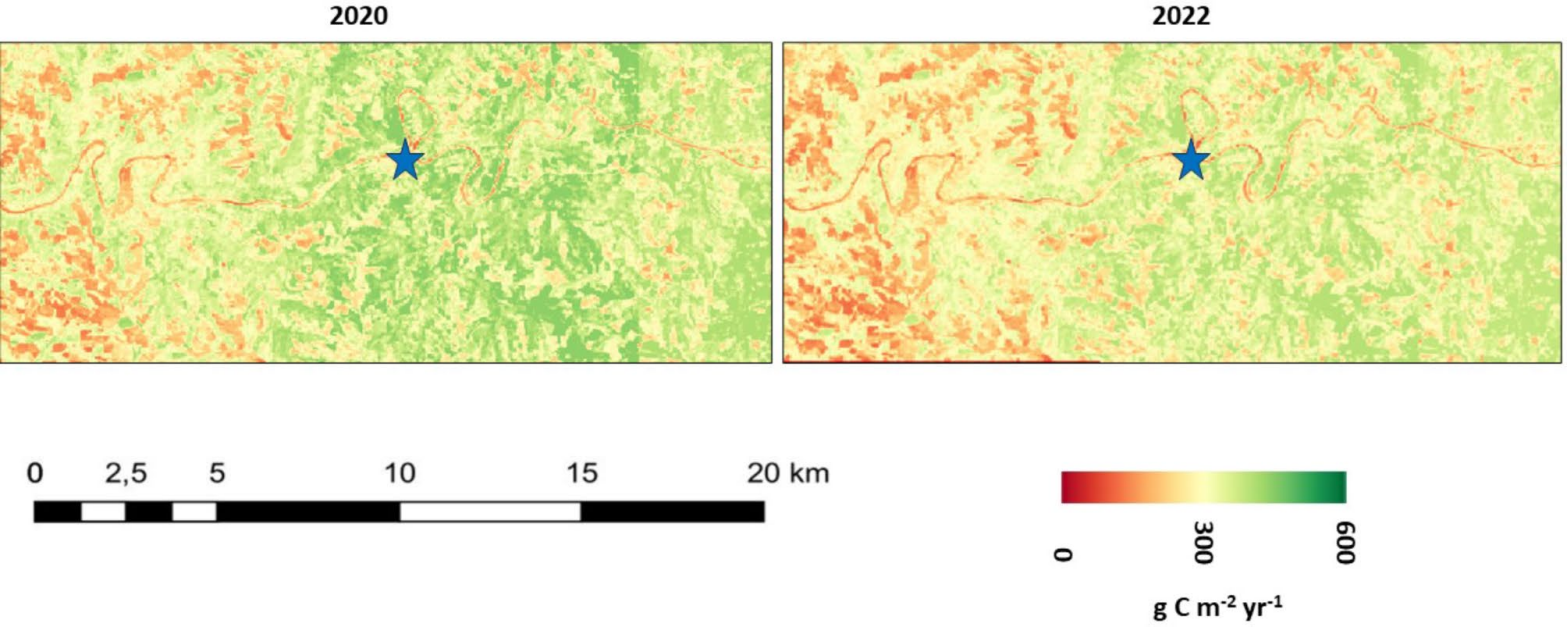
CASA model NPP at 30-m Landsat pixel resolution predicted for the growing seasons of 2020 (left) and 2022 (right) for the Garonne River basin drainages of Le Tarn River to the east of Albi. Location of blue star symbol is 43.94^o^ N, 2.38^o^ E near the city of Ambialet

Localities within the Garrone River basin (example in Fig. 9) where 2020/2021 growing season NPP was reduced by 50% or more by 2022 drought conditions included:

- Lower Aveyron and Le Tarn River sub-basins around the city of Albi. Principal crops grown in these valleys are maize, wheat, fruit trees, and alfalfa, much of which is irrigated [36].
- Lower Ger and Gimone River sub-basins around the city of Auch, flowing into the Garonne near Agen.

The Po River is Italy’s longest, flowing 650 km through the northern Piedmont region and along borders of the Lombardy, Emilia-Romagna, and Veneto regions. The river’s 46,000 km^2^ drainage basin forms the Po Valley, Italy’s most important agricultural area, hosting a population of 17 million, a third of Italy’s total population. Nonetheless, the valley’s surface runoff water is of little use for drinking and household uses, with flows being unreliable, often destructive in floods, and heavily polluted by sewage and fertilizers [37]. The Po River’s main uses are for hydro-electric power, irrigation, and industrial transport. The principal crops grown are wheat, maize, hay, barley, sugar beets, grapes, and rice.

**Fig. 10:**
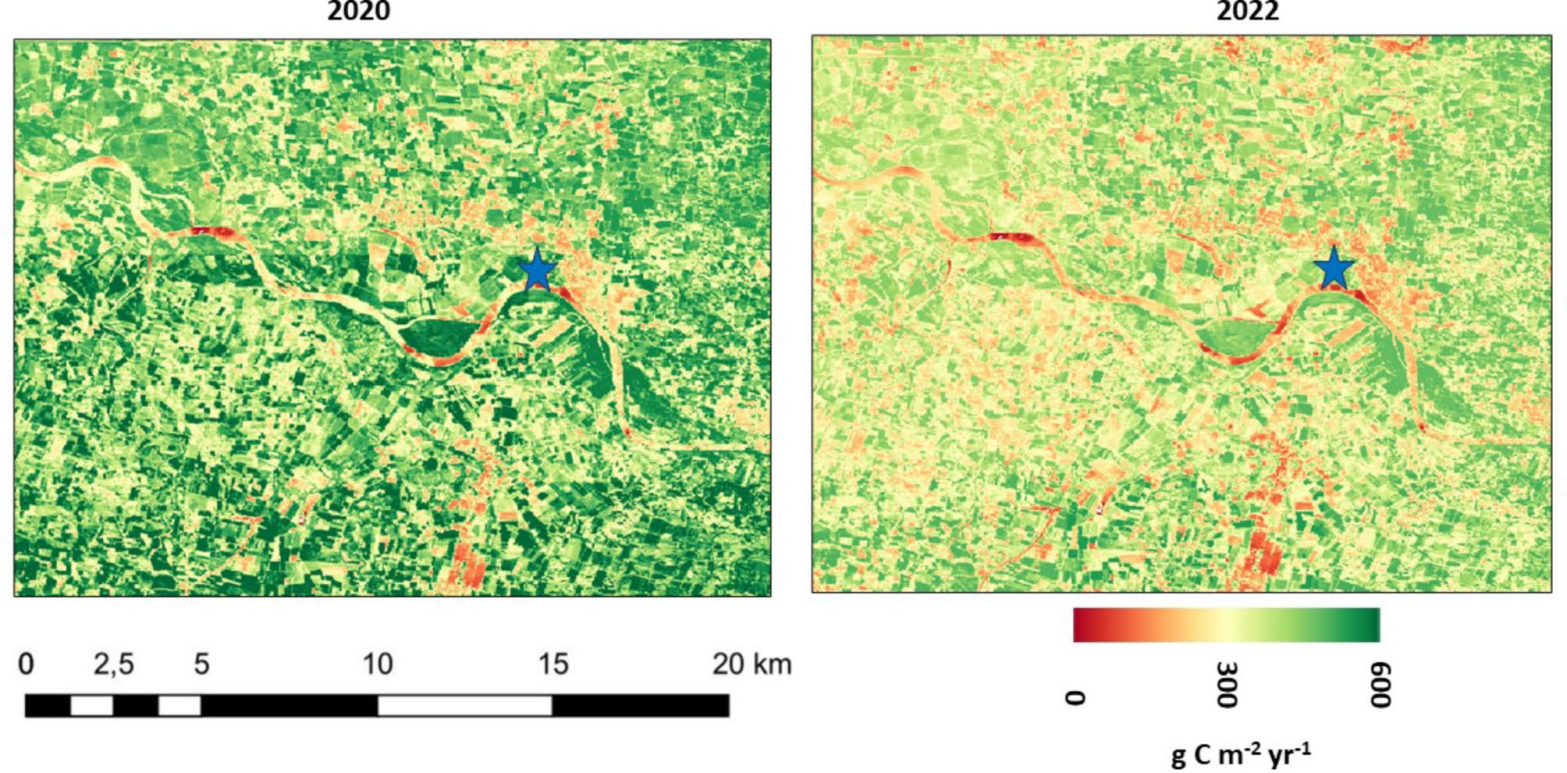
CASA model NPP at 30-m Landsat pixel resolution predicted for the growing seasons of 2020 (left) and 2022 (right) for the Po River basin drainages north of Parma. Location of blue star symbol is 44.99^o^ N, 10.41^o^ E near the city of Casalmaggiore

Localities within the Po River basin (example in Fig. 10) where 2020/2021 growing season NPP was reduced by 50% or more by 2022 drought conditions included:

- Lower Tanaro River sub-basins north of the city of Genova. Principal crops grown in these lowlands around the city of Alessandria are maize and wine grapes;
- Lower Taro and Parma River sub-basins around the city of Fidenza. Principal crops grown in these lowlands are wheat, tomatoes, and alfalfa as forage for dairy cattle;
- Lower Reno River sub-basins southeast of the city of Bologna. The principal crop grown is maize, consuming 30% of irrigation water supplied in the Reno River valleys.

## Discussion

The results from this CASA ecosystem modeling study imply that there have been major variations in plant production levels in Western Europe over the period of 2015 to 2022, due primarily to the most extreme and widespread droughts recorded on the continent in centuries. The summer growing season of 2018 showed the highest terrestrial plant growth over the study period in France, Greece, Italy, Portugal, and Spain. Gradual increases in average predicted NPP across these countries from 2015 to 2021 were followed by typical declines of 25–60% of growing season NPP from 2021 to 2022.

Countries of the Mediterranean region, namely Spain, France, Italy, and Croatia, experienced the most severe loss of plant production in 2022 and much of it in the important agricultural valleys of these nations. The CASA model estimated that agricultural lands within these countries showed an average loss of growing season NPP in 2022 of -25% (compared to the 2020 and 2021 growing seasons), although numerous sub-basins of the Ebro, Garrone and Po River drainages showed NPP declines in excess of -50% in 2022. Publications and reports issued by European-focused governmental agencies are reviewed in the section that follows to substantiate the CASA model NPP predictions presented in this paper.

In a study of extreme weather events in Europe over the past 50 years [38], reported that historical droughts and heatwaves have reduced average European cereal yields by 9% and 7%, respectively, while observing a wide range of responses (inter-quartile ranges of + 2% to − 23%). Non-cereal crop yields declined by 3.8% and 3.1% during the same period of weather events. This analysis found that the severity of drought and heatwave impacts on crop production have tripled over the past 50 years in Europe and drought-related cereal production declines were shown to intensify by more than 3% annually.

In 2016 and 2017, much of western Europe experienced a severe drought event [7]. In 2018, drought impacted plant cover from Switzerland into the Benelux and Germany, and from the Czech Republic into Sweden and Finland [39]. Notable declines in crop yields had been reported, especially for cereals, olives, tomatoes, wine-grapes and almonds in Spain and Italy [40]. Likewise, relatively low levels of plant production were estimated by the CASA model in 2016 and 2017 for France, Spain, Portugal, Italy, and Greece (Fig. 4).

From recent country-specific perspectives, the National Economic Accounts for agriculture [41] estimated that overall crop production in Spain declined by 13.6% in 2022; specifically, water storages resulted in lower yields for grains (-24%), fruits (-21%), forage plants (-18%), vegetables (-8%) and potatoes (-7%). According to France’s Ministry of Agriculture, crop irrigation accounts for 45% of the country’s water supply, followed by 31% for power generation and 21% for drinking water. In 2022, crop condition indices in France declined steeply during July [42]. Summer corn production in France was reported to have declined in 2022 by 31% and wheat production declined by 7% (compared to 2021).

According to Italy’s largest agricultural association, Coldiretti (Confederazione Nazionale Coltivatori Diretti), water shortages in 2022 caused a 10% decline in Italy’s agricultural production compared to previous years, although other reports (www.forbes.com/sites/carlieporterfield/2022/07/13/italian-drought-puts-one-third-of-national-agriculture-production--like-tomatoes-and-olive-oil--at-risk/) put losses of crop production in 2022 much higher (> 30%) in Italy’s northern region.

The causes for these extreme weather impacts in Northern Italy were explained in an analysis of more than 200 years of flow data the Po River outlet by Montanari et al.[43]. These authors reported that the 2022 drought was the worst such event in Italy’s recorded climate history, with 30% lower river flows than the second worst drought on record, and an estimated 600-year return interval. The 2022 decline in summer Po River flows (-4.1 m^3^ s^− 1^) was attributed to a combination of changes in precipitation timing and water supply, namely in a decline of snow fraction (-0.6% yr^− 1^) and snowmelt rate (-0.18 mm da^− 1^), and an increasing evaporation rate (+ 0.013 km^3^ yr^− 1^). These authors concluded that recent declines in snowfall in the Alps have very likely contributed to the reduction in the Po River flows in June and July. Warming temperatures in southern Europe’s alpine elevations have resulted in more precipitation falling as rain rather than snow, advancing a higher yearly fraction of snowmelt and runoff into the spring months.

It is worthwhile to mention some limitations of the modeling methods presented in this study, namely related to the paucity of information available from local sources in Western Europe on the types of crops cultivated in the Ebro, Garrone, and Po River basins and how they are irrigated and rotated from year to year. As pointed out by Jay et al. (2016) in a CASA validation study, the model is sometimes (but not always) less accurate for cropland NPP than forest NPP, and that difference was derived mainly from the local cropping data being incomplete to provide CASA will all the inputs it requires to simulate droughts and managed crop rotations impacting NPP.

## Conclusions

CASA model predictions from 2015 to 2022 showed variations between high and low periods in growing season NPP totals in most countries of Western Europe. For the total land coverage of France, Greece, Italy, Portugal, and Spain, 2018 was the year with the highest terrestrial plant growth over the study period, whereas 2017 and 2019 were the years with the highest summed NPP across the UK, Germany, and Croatia. For most of Western Europe, 2022 was the year predicted with the lowest summed plant growth. Annual precipitation in most countries of Western Europe progressively declined from a high average rate of > 90 cm y^− 1^ in 2018 to a low average precipitation rate of < 80 cm y^− 1^ in 2022. The CASA model predicted decreased growing season NPP of between − 25 and − 60% across all of Spain, southern France, and northern Italy from 2021 to 2022, and much of that production loss was detected in the important agricultural valleys of these counties.

## Data Availability

The CASA model source code used in this study and CASA model NPP output geotiff files are all available for download at the NASA-CASA Github repository in version 2024, openly accessible at https://zenodo.org/records/10525125 [44] (Potter and Pass, 2024, 10.5281/zenodo.10525125). There are no access limitations nor licensing conditions for the use of this software.
